# Pachypodol, a Methoxyflavonoid Isolated from *Pogostemon cablin* Bentham Exerts Antioxidant and Cytoprotective Effects in HepG2 Cells: Possible Role of ERK-Dependent Nrf2 Activation

**DOI:** 10.3390/ijms20174082

**Published:** 2019-08-21

**Authors:** Eun Kyung Kim, Ji Hoon Kim, Soyeon Jeong, Yong Won Choi, Hyun Jung Choi, Chul Young Kim, Young-Mi Kim

**Affiliations:** Department of Pharmacy, College of Pharmacy and Institute of Pharmaceutical Science and Technology, Hanyang University, Ansan, Gyeonggi-do 15588, Korea

**Keywords:** antioxidants, ERK, hepatocyte protection, Nrf2, pachypodol, *Pogostemon cablin*

## Abstract

Oxidative stress has been implicated in the pathogenesis of many diseases including chronic liver diseases. Nrf2 is a master transcriptional factor regulating the induction of cellular antioxidant defense systems. Here, the Nrf2-activating effect of the crude methanol extract of dried leaves of *Pogostemon cablin* Bentham was demonstrated by measuring the antioxidant response element (ARE)-driven luciferase activity and pachypodol, 4′,5-dihydroxy-3,3′,7-trimethoxyflavone, was isolated by bioactivity-guided fractionation and further separation using chromatographic techniques. To our knowledge, this is the first study to evaluate the antioxidant and cytoprotective effects of pachypodol in HepG2 cells as well as the underlying molecular mechanisms. Indeed, pachypodol protected HepG2 cells from cell death caused by *tert*-butylhydroperoxide-induced oxidative stress and also attenuated ROS production. The ability of pachypodol to activate Nrf2/ARE pathway was further confirmed by observing Nrf2 expression in nuclear fraction, mRNA levels of Nrf2 target antioxidants, and cellular glutathione content in HepG2 cells. Extracellular signal-regulated kinase (ERK) is one of the important kinases involved in Nrf2 activation. Pachypodol increased ERK phosphorylation and ERK inhibition by PD98059 totally abrogated the increase in ARE luciferase activity, nuclear Nrf2 accumulation and mRNA levels of antioxidant enzymes by pachypodol. In conclusion, pachypodol isolated from *P. cablin* can protect hepatocytes from oxidative injury, possibly mediated by enhancing endogenous antioxidant defense system through ERK-dependent Nrf2 activation.

## 1. Introduction

Reactive oxygen species (ROS) can play an important role in the physiological regulation of diverse cell functions, but excessively generated ROS can damage the structure and function of major cellular components, such as proteins, lipids and DNA [1]. Oxidative stress resulting from the imbalance between oxidants and antioxidants has been implicated in the process of aging and the development of many diseases including cancer and neurodegenerative, chronic inflammatory, cardiovascular and metabolic diseases [1,2]. The importance of oxidative stress in the pathogenesis of various diseases suggests the potential of antioxidants for the prevention and treatment of these diseases.

NF-E2-related factor-2 (Nrf2) is a master transcriptional factor that regulates the expression of a variety of cytoprotective genes including antioxidant and detoxifying enzymes, and antiapoptotic proteins by binding to antioxidant response element (ARE) [3]. Upon exposure to oxidative stress or various antioxidants including phytochemicals, Nrf2 is dissociated from Nrf2-Kelch-like ECH-associated protein 1 (Keap1)-Cul3 complex in the cytoplasm and translocates into the nucleus [4]. Diverse mechanisms affecting the transcriptional activity of Nrf2 have been reported [4,5]. Since the Nrf2/ARE signaling pathway is a key component of the coordinated induction of cellular antioxidant defense systems to maintain redox homeostasis [4], beneficial effects of pharmacological Nrf2 activators have been studied in many diseases in which oxidative stress is involved [6,7,8].

*Pogostemon cablin* Bentham (also known as patchouli) is an aromatic herb and has been widely used to remove dampness, alleviate summer heat, and stimulate appetite in Oriental medicine [9,10]. Several biological effects of extracts of *P. cablin* have been previously reported, which include antiemetic, antimicrobial, antiviral, antimutagenic, antioxidant, anti-inflammatory, and analgesic effects and gastrointestinal protective activities [9,11,12,13,14,15,16]. Various phytochemical compounds including patchouli alcohol, pogostone, and methoxylated flavonoids were identified in the herb of *P. cablin* [9,17]. Most of the previous studies have focused on exploring the pharmacological effects of the patchouli essential oil, a major composition of the leaves of *P. cablin* [18,19,20] and the main volatile constituents contained in patchouli oil, such as patchouli alcohol, pogostone, and patchoulenes [21,22,23,24,25,26]. Relatively few studies have been conducted to investigate the pharmacological activities of the non-volatile constituents of *P. cablin*.

In this study, we examined the Nrf2-activating effects of the crude methanol extract of dried leaves of *P. cablin* Bentham and its solvent fractionations in HepG2 cells, and isolated and identified the two major Nrf2-activating components, pachypodol (4′,5-dihydroxy-3,3′,7-trimethoxyflavone) and eriodictyol 3′,7-dimethyl ether (4′,5-dihydroxy-3′,7-dimethoxyflavanone) by centrifugal partition chromatography (CPC), an efficient procedure for the bioactivity-guided isolation of natural compounds. To date, only a few studies have been conducted to elucidate the pharmacological activities of these two methoxyflavonoids. To our knowledge, this is the first study to evaluate the antioxidant and cytoprotective effects of pachypodol in HepG2 cells as well as the underlying molecular mechanisms.

## 2. Results

### 2.1. Bioactivity-Guided Isolation of Pachypodol and Eriodictyol 3′,7-Dimethyl Ether from P. cablin

**Figure 1:**
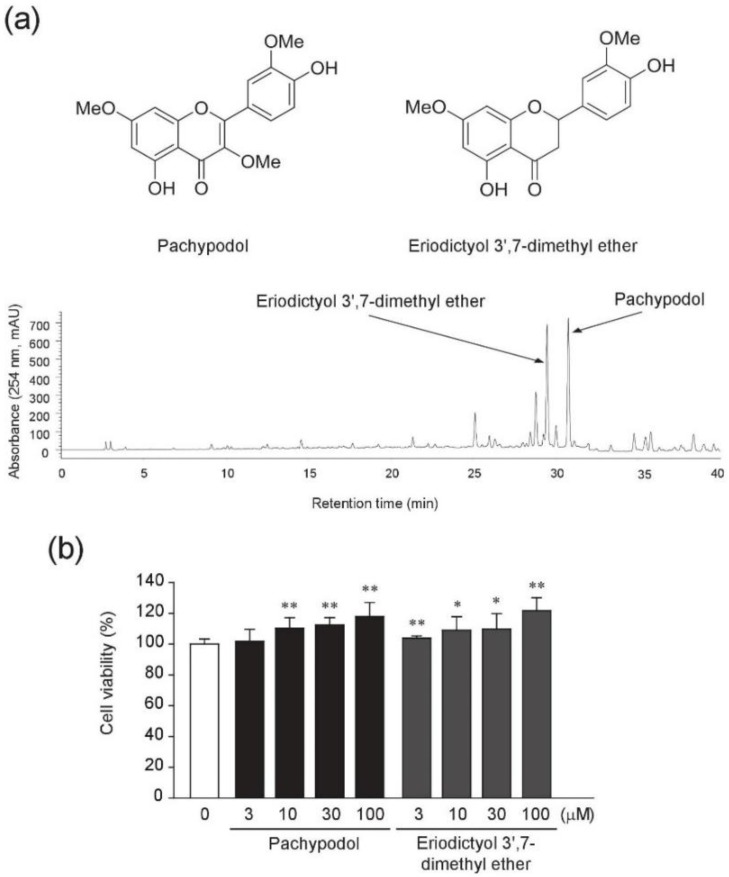
Chemical structure and cytotoxicity of pachypodol and eriodictyol 3′,7-dimethyl ether in HepG2 cells. (**a**) Chemical structures and an HPLC chromatogram of the crude methanol extract from the dried leaves of *Pogostemon cablin* Bentham; (**b**) HepG2 cells were exposed to each compound (3–100 μM) for 24 h after overnight serum starvation and the cell viability was measured by an MTT colorimetric assay. Data represent the mean ± S.D. (*n* = 8). * *p* < 0.05, ** *p* < 0.01 (compared with the dimethyl sulfoxide (DMSO)-treated vehicle control); MTT, 3-(4,5-dimethylthiazol-2-yl)-2,5-diphenyltetrazolium bromide.

The ARE-driven luciferase activities were measured to identify the major Nrf2-activating components by the bioactivity-guided isolation. The scheme for the extraction, fractionation and isolation of major active compounds from the crude methanol extract of dried leaves of *P. cablin* Bentham and HPLC chromatogram of crude extract are provided in Figure S1 and Figure 1a, respectively. In addition, HPLC chromatograms of *n*-hexane fraction and CPC sub-fractions are shown in supplemental figure (Figure S2a). ARE luciferase activities were determined in the lysates of HepG2 cells stably transfected with pGL4.37 plasmid which contains four copies of an ARE. Treatment with 30 μg/mL of *n*-hexane fraction and its CPC sub-fraction 2 showed much higher increases in ARE-luciferase activities (Figure S2b). Subsequently, two major compounds which account for most of the CPC sub-fraction 2 as shown by HPLC chromatogram (Figure S2a) were isolated and identified as pachypodol and eriodictyol 3′,7-dimethyl ether through direct comparison of their spectroscopic data with those previously given in the literature [27,28] (Figures S3 and S4).

### 2.2. Effects of Pachypodol and Eriodictyol 3′,7-Dimethyl Ether on the Nrf2-ARE Pathway and t-BHP-Induced Cell Death

**Figure 2:**
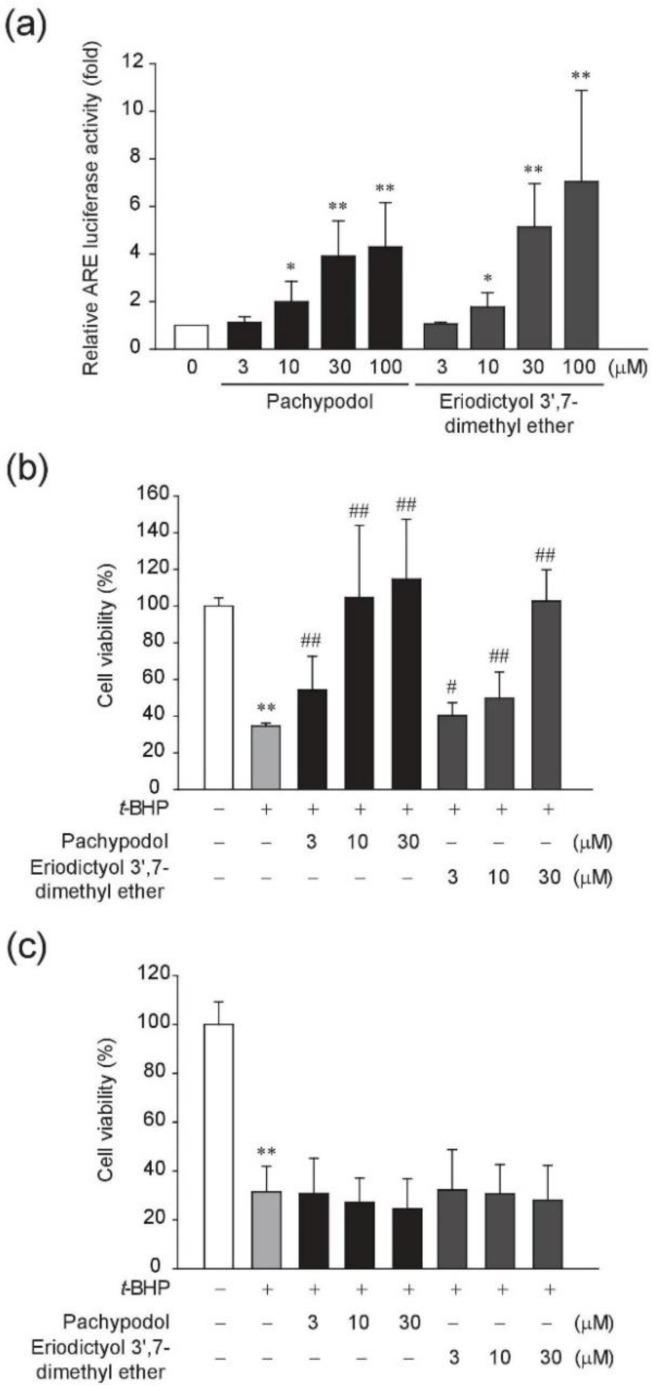
Effects of pachypodol and eriodictyol 3′,7-dimethyl ether on the Nrf2-antioxidant response element (ARE) pathway and *t*-BHP-induced cell death. (**a**) The ARE-luciferase activity was measured in the lysates of HepG2 cells stably transfected with pGL4.37 plasmid. Cells were treated with each compound for 12 h. Data represent the mean ± S.D. (*n* = 5); (**b**,**c**) HepG2 cells were preincubated with the indicated concentrations of each compound for 12 h (**b**) or 1 h (**c**) and subsequently exposed to 500 μM *t*-BHP for 4 h. Cell viability was determined by an MTT assay. Data represent the mean ± S.D. (*n* = 8). * *p* < 0.05, ** *p* < 0.01 (compared with the DMSO-treated vehicle control); ^#^ *p* < 0.05, ^##^ *p* < 0.01 (compared with the group treated with *t*-BHP alone); ARE, antioxidant response element; *t*-BHP, *tert*-butylhydroperoxide.

We next investigated whether these two methoxyflavonoids contribute to the increase in ARE- luciferase activity by CPC sub-fraction 2. First, their cytotoxic effects were evaluated in HepG2 cells. Both pachypodol and eriodictyol 3′,7-dimethyl ether showed no cytotoxicity when treated at the concentrations of 3–100 μM for 24 h (Figure 1b). As shown in Figure 2a, they enhanced the ARE-luciferase activities in a concentration-dependent manner and statistically significant increases were detected from the concentration of 10 μM. Since it has been reported that dimethyl sulfoxide (DMSO), used as a vehicle in this study can activate Nrf2 [29], we examined the effect of DMSO on the Nrf2-ARE pathway in HepG2 cells. When exposed to 0.1%–0.2% DMSO, the ARE- luciferase activity was not different from that in the non-treated cells, but ~1.2-fold and 1.8-fold increase was observed in 0.4% and 0.8% DMSO-treated HepG2 cells, respectively (Figure S5). Considering any possible effect of the vehicle, the same amount of DMSO (0.1% in this study) was used as a vehicle control for each compound, and the result was compared with that of DMSO-treated vehicle control. 

With regard to their ability to activate Nrf2, we further tested whether they can protect cells from oxidative stress-induced death in HepG2 cells. *tert*-Butylhydroperoxide (*t*-BHP) is often used to cause cellular oxidative stress through its conversion to free radicals [30,31]. In this study, *t*-BHP alone reduced cell viability to ~35% of control levels, but the pretreatment with either pachypodol or eriodictyol 3′,7-dimethyl ether for 12 h significantly prevented oxidative stress-induced cell death, which was concentration-dependent (Figure 2b). By contrast, the cytoprotective effect was not seen by only 1 hour-pretreatment of each compound (Figure 2c), suggesting that the longer pretreatment times for the preceding activation of endogenous antioxidant defense systems are required in our experimental conditions. In the following experiments to further elucidate the antioxidant effects and to explore the underlying molecular mechanism, we used pachypodol which showed more potent cytoprotective effects in HepG2 cells.

### 2.3. Effects of Pachypodol on the t-BHP-Induced ROS Production and the Intracellular Antioxidant System

**Figure 3:**
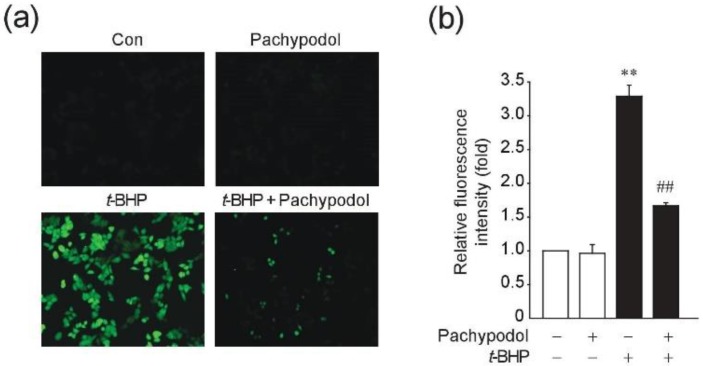
Effects of pachypodol on the ROS production. HepG2 cells were exposed to 500 μM *t*-BHP for 30 min following the 12 h-pretreatment of pachypodol. The intracellular ROS generation was determined by fluorescence microscopy after cells were stained with CM-H_2_DCFDA (10 μM). The representative images (**a**, 20×) and the quantified fluorescence intensities (**b**) are shown. Data represent the mean ± S.D. (*n* = 3). ** *p* < 0.01 (compared with the DMSO-treated vehicle control); ^##^ *p* < 0.01 (compared with the group treated with *t*-BHP alone); CM-H_2_DCFDA, 5-(and-6)-chloromethyl-2′,7′-dichlorodihydrofluorescein diacetate acetyl ester.

We next determined the effect of pachypodol on the cellular ROS levels in HepG2 cells. Only 30 minute-exposure to *t*-BHP increased the intracellular ROS level by ~3-fold (Figure 3) and pretreatment with pachypodol clearly decreased it, which was expected from its cytoprotective effect.

**Figure 4:**
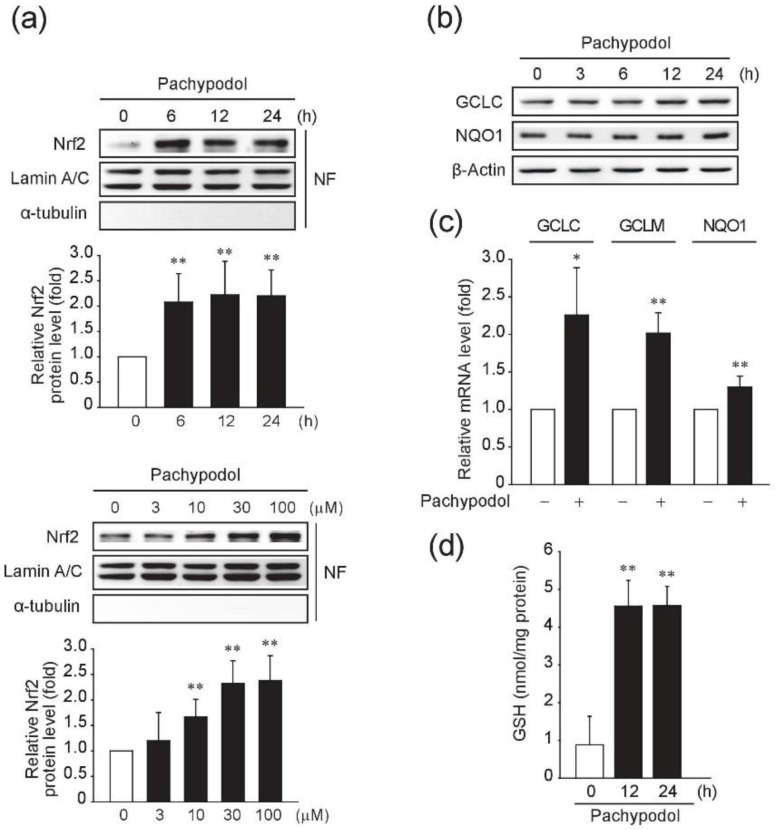
Induction of the intracellular antioxidant system by pachypodol. (**a**) Western blotting was performed to analyze the nuclear Nrf2 protein levels in HepG2 cells exposed to 30 μM pachypodol for the indicated times (upper) or treated with increasing concentrations of pachypodol for 6 h (lower). Lamin A/C (a nuclear protein) was used as a loading control and the purity of nuclear fraction was verified by immunoblotting for α-tubulin (a cytosolic protein). Bands were quantified by densitometric scanning. Nuclear Nrf2 levels were normalized to those of lamin A/C. Data represent the mean ± S.D. (*n* = 6 or 4); (**b**) The antioxidant enzyme expression was determined by immunoblotting in the lysates of HepG2 cells treated with 30 μM pachypodol for the indicated times; (**c**) The mRNA levels of GCLC, GCLM and NQO1 were measured by real-time PCR analysis in HepG2 cells exposed to 30 μM pachypodol for 6 h. Data represent the mean ± S.D. (*n* = 3–4); (**d**) The intracellular GSH contents were determined in HepG2 cells after exposure to 30 μM pachypodol for the indicated times. Data represent the mean ± S.D. (*n* = 3). * *p* < 0.05 ** *p* < 0.01 (compared with the DMSO-treated vehicle control); GCLC, catalytic subunit of glutamate-cysteine ligase; GCLM, modifier subunit of glutamate-cysteine ligase; GSH, glutathione; NF, nuclear fraction; NQO1, NAD(P)H:quinone oxidoreductase 1.

In addition to the enhanced ARE-driven luciferase activity by pachypodol which was described earlier, we determined nuclear translocation of Nrf2 to further confirm its ability to activate Nrf2. Obvious nuclear accumulation of Nrf2 was seen after 6 hour-exposure to pachypodol (Figure 4a, upper). Concentration-dependent increase in nuclear Nrf2 levels was also observed (Figure 4a, lower). Furthermore, the representative Nrf2-controlled antioxidant genes including catalytic subunit of glutamate-cysteine ligase (GCLC), modifier subunit of glutamate-cysteine ligase (GCLM), and NAD(P)H:quinone oxidoreductase 1 (NQO1) were upregulated by pachypodol at both the mRNA and protein levels (Figure 4b,c). Considering that the glutamate-cysteine ligase (GCL) comprising GCLC and GCLM is responsible for the rate-limiting step in the biosynthesis of glutathione (GSH), we measured the intracellular GSH contents. Indeed, the cellular GSH level dramatically increased (~5-fold) in HepG2 cells treated with pachypodol for 12 h (Figure 4d), which is consistent with the upregulation of GCLC and GCLM by pachypodol.

### 2.4. Role of ERK Activation in the Nrf2 Activation and Cytoprotection by Pachypodol

**Figure 5:**
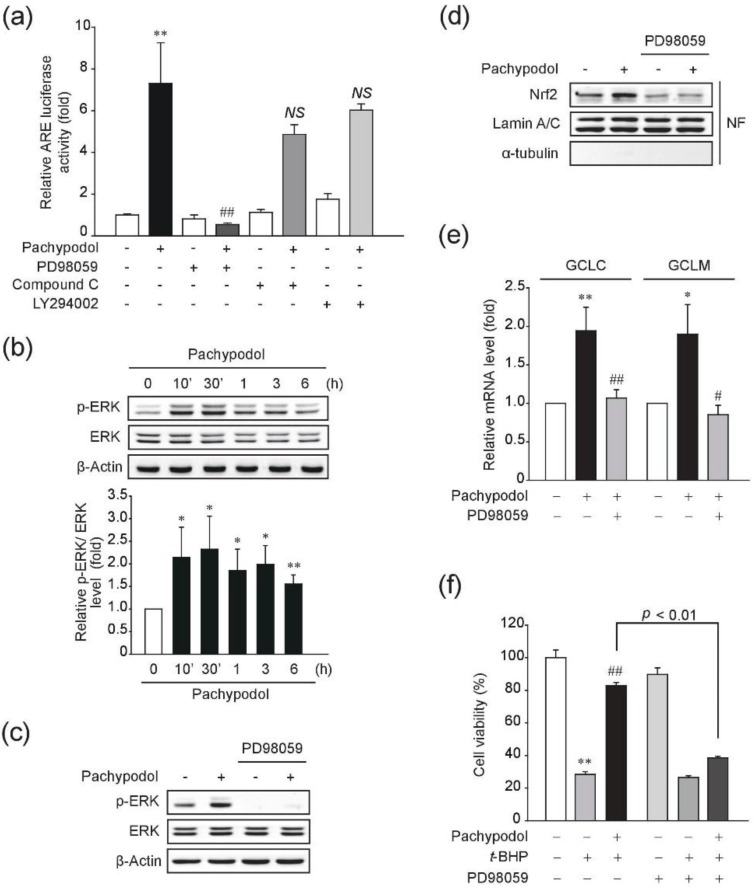
Role of ERK activation in the Nrf2 activation and cytoprotection by pachypodol. (**a**) The ARE-luciferase activity was measured in the lysates of pGL4.37 plasmid stably transfected HepG2 cells that had been exposed to pachypodol (30 μM, 12 h) after pretreatment with 10 μM PD98059, a MEK inhibitor, 1 μM Compound C, an AMPK inhibitor, or 10 μM LY294002, a PI3K inhibitor for 1 h; (**b**) The time-dependent ERK phosphorylation by the treatment of 30 μM pachypodol in HepG2 cells were assessed by Western blot analysis. Bands were quantified by densitometric scanning. The expression levels of phosphorylated ERK were normalized to those of its total form; (**c**) ERK inhibition by PD98059 was verified by immunoblotting in the lysates of HepG2 cells exposed to pachypodol (30 μM, 30 min) after 1 h pretreatment with PD98059 (10 μM); (**d**,**e**) Nuclear Nrf2 levels or GCLC and GCLM mRNA levels were determined in HepG2 cells exposed to pachypodol (30 μM, 6 h) after 1 h pretreatment with PD98059 (10 μM). Lamin A/C (a nuclear protein) was used as a loading control and the purity of nuclear fraction was verified by immunoblotting for α-tubulin (a cytosolic protein); (**f**) HepG2 cells were treated as described in the legend to Figure 2b in the presence or absence of PD98059 (10 μM). Cell viability was assessed by an MTT assay. Data represent the mean ± S.D. (*n* = 3–4). * *p* < 0.05, ** *p* < 0.01 (compared with the DMSO-treated vehicle control); ^#^ *p* < 0.05, ^##^ *p* < 0.01 (compared with the pachypodol-treated group or *t*-BHP alone); AMPK, AMP-activated protein kinase; GCLC, catalytic subunit of glutamate-cysteine ligase; GCLM, modifier subunit of glutamate-cysteine ligase; MEK, mitogen-activated protein kinase kinase; NF, nuclear fraction; *NS*, not significant; PI3K, phosphatidylinositol 3-kinase.

Next, we further investigated upstream signaling pathways of the Nrf2/ARE activation by pachypodol. Several kinases have been reported to play an important role in the regulation of Nrf2 transcriptional activity [5]. Among them, we focused on the role of extracellular signal-regulated kinase (ERK), phosphoinositide 3-kinase (PI3K)/Akt, and AMP-activated protein kinase (AMPK) which have been known to be frequently involved in the activation of Nrf2/ARE system stimulated by many phytochemicals [32,33,34,35]. To investigate which upstream kinases are responsible for the Nrf2 transcriptional activation by pachypodol, a specific inhibitor of each kinase signaling pathway was pretreated. As shown in Figure 5a, ERK inhibition by PD98059, a mitogen-activated protein kinase kinase (MEK) inhibitor, totally abrogated the increase in the ARE luciferase activity by pachypodol, whereas the inhibition of either AMPK or PI3K/Akt signaling pathway had no significant effect. In this regard, we examined if pachypodol can affect the phosphorylation status of ERK in HepG2 cells. Indeed, ERK phosphorylation clearly increased from 10 min of exposure to pachypodol, which lasted up to 6 h (Figure 5b). ERK inhibition by the treatment with 10 μM PD98059 was verified in Figure 5c. To further investigate the role of ERK activation in pachypodol-induced transcriptional activation of Nrf2, nuclear Nrf2 levels were observed in the presence of PD98059. As shown in Figure 5d, pachypodol-stimulated nuclear accumulation of Nrf2 was abrogated by ERK inhibition. In line with this result, the increase in GCLC and GCLM mRNA levels by pachypodol was also completely blocked by ERK inhibition (Figure 5e). Taken together, these results demonstrate the essential role of ERK activation in pachypodol-induced transcriptional activation of Nrf2.

Furthermore, we assessed the cytoprotective effect of pachypodol against *t*-BHP-induced oxidative damage in the presence of PD98059. As already demonstrated in Figure 2b, pachypodol markedly prevented oxidative stress-induced cell death, but ERK inhibition by PD98059 dramatically attenuated the cytoprotective effect of pachypodol (Figure 5f). These results suggest that pachypodol protects cells from oxidative stress-induced cell death through ERK activation. 

## 3. Discussion

To date, few studies investigating the cytoprotective and antioxidant effects of *P. cablin* are available. Kim et al. have reported the protective effect of water extract of *P. cablin* against hydrogen peroxide (H_2_O_2_)-induced oxidative injury in the human neuroglioma cell line, possibly mediated by scavenging ROS [14]. Main components in the water extract responsible for its cytoprotective effect have not been identified in their study. Direct effects of this water extract of *P. cablin* on the Nrf2/ARE pathway have not been evaluated, but the Nrf2-activating effects of pogostone and β-patchoulene, biologically active constituents of the essential oil from *P. cablin* have been reported in the studies investigating their protective activities against lipopolysaccharide (LPS)-induced acute lung injury in mice [25,26]. Pretreatment with either pogostone or β-patchoulene enhanced the expression of antioxidant genes along with the nuclear accumulation of Nrf2 in the lung tissue compared with those in the group treated with LPS alone.

Here, we for the first time demonstrated that crude methanol extract of dried *P. cablin* and its *n*-hexane fraction can directly stimulate the Nrf2/ARE pathway. We also purified two major Nrf2-activating compounds, pachypodol and eriodictyol 3′,7-dimethyl ether from *n*-hexane extract of *P. cablin* by the bioactivity-guided isolation. Pachypodol belongs to the major non-volatile constituents of *P. cablin* [9], but there have been only a few studies exploring its pharmacological activities. The antiemetic [11] and antimutagenic [13] effects, and cytotoxic effects in several cancer cell lines [27,36] have been reported. Relatively in-depth studies have been reported on its selective antiviral activities against picornaviruses, such as rhinovirus, coxsackievirus, and poliovirus [37,38]. Recently, phosphatidylinositol 4-kinase III beta has been suggested as a major target of pachypodol for its anti-picornavirus effects [39]. However, to the best of our knowledge, pharmacological effects of pachypodol in the liver have never been studied. In this study, we first report the antioxidant and cytoprotective effects of pachypodol in hepatocytes, which involve the Nrf2/ARE pathway.

The Nrf2-activating ability of pachypodol was first verified by the enhanced ARE-driven luciferase activity in HepG2 cells and further confirmed by the nuclear accumulation of Nrf2 as shown by Western blot analysis. Furthermore, its ability to enhance the intracellular antioxidant defense system has been demonstrated by the induction of Nrf2-regulated antioxidant enzymes and a marked increase in the level of GSH, a major endogenous non-protein antioxidant. Considering that the intracellular GSH depletion causes oxidative damage [40] and GSH level is dramatically reduced by *t*-BHP treatment as shown in our previous study [32], the Nrf2-dependent endogenous antioxidant defense system enhanced by the treatment of pachypodol may contribute to its protective effects against *t*-BHP-induced oxidative stress and subsequent hepatocyte cell death. 

Several kinases including ERK, PI3K/Akt, AMPK, PKCδ, PERK, GSK3β, and Fyn kinase have been known to be involved in various mechanisms regulating the transcriptional activation of Nrf2 [5,41]. In our study, ERK has been identified to be the main contributor to pachypodol-induced Nrf2/ARE activation based on our results that ERK inhibition completely blocked the increase in ARE-luciferase activity, nuclear Nrf2 expression and mRNA expression of GCLC and GCLM by pachypodol. This essential role of ERK activation was further supported by pachypodol-stimulated ERK phosphorylation. ERK has been reported to facilitate the dissociation of the Keap1-Nrf2 complex and nuclear translocation of Nrf2 [5,42,43]. The importance of ERK signaling pathway in the regulation of Nrf2 activation has been also demonstrated in many previous studies investigating pharmacological effects of phytochemicals including epigallocatechin gallate, nectandrin B, sulforaphane, and quercetin [32,33,35]. 

Since liver is a main site of the metabolism of xenobiotics and endogenous compounds, it is constantly exposed to ROS. Generally, it has been accepted that the sustained and excessive generation of ROS in the liver is closely associated with the pathogenesis of most liver diseases, thus suggesting the therapeutic potential of an Nrf2-mediated inducer of endogenous antioxidant defense system in the development and progression of various liver diseases [6,7].

In conclusion, pachypodol, a methoxyflavonoid isolated from *P. cablin* Bentham can protect hepatocytes from oxidative injury, possibly mediated by enhancing endogenous antioxidant defense system through ERK-dependent Nrf2 activation. This result suggests the therapeutic potential of pachypodol in common chronic liver diseases, such as nonalcoholic fatty liver disease, nonalcoholic steatohepatitis, and alcoholic liver disease.

## 4. Materials and Methods 

### 4.1. Materials

Anti-GCLC and anti-NQO1 antibodies, anti-Nrf2 antibody, and anti-α-tubulin antibody were provided by Abcam (Cambridge, MA, USA), Santa Cruz Biotechnology (Santa Cruz, CA, USA), and Rockland Immunochemicals (Gilbertsville, PA, USA), respectively. Antibodies against phospho-ERK, ERK, and lamin A/C were purchased from Cell Signaling Technology (Beverly, MA, USA). 5-(and-6)-chloromethyl-2′,7′-dichlorodihydrofluorescein diacetate acetyl ester (CM-H_2_DCFDA), PD98059, and Compound C were obtained from Invitrogen (Carlsbad, CA, USA), Millipore (Billerica, MA, USA), and Calbiochem (SanDiego, CA, USA), respectively. Anti-β-actin antibody, 3-(4,5-dimethylthiazol-2-yl)-2,5-diphenyltetrazolium bromide (MTT), *tert*-butylhydroperoxide (*t*-BHP), LY294002, and other reagents were supplied by Sigma-Aldrich Co. (St. Louis, MO, USA).

### 4.2. Plant Material and Extraction, and Isolation of Pachypodol

The aerial parts of *P. cablin* were purchased from the Kyungdong oriental herbal market, Seoul, Korea, in September 2016, and identified by one of author (Dr. CY Kim). A voucher specimen was deposited in the Herbarium of the College of Pharmacy, Hanyang University (HYUP-PC-001). The dried *P. cablin* (200 g) was extracted in 500 mL methanol three times for 3 h under reflux. The methanol solution was concentrated by rotary evaporator to obtain 67 g of extract. The extract was suspended in water and successively partitioned with *n*-hexane and ethyl acetate to give *n*-hexane (36.2 g), ethyl acetate (13.7 g) and aqueous (16.1 g) extracts. Pachypodol (4′,5-dihydroxy-3,3′,7-trimethoxyflavone) and eriodictyol 3′,7-dimethyl ether (4′,5-dihydroxy-3′,7-dimethoxyflavanone) were isolated and purified from *n*-hexane extract of *P. cablin* by CPC and preparative HPLC as described in the legend to Figure S2. The chemical structures were confirmed by ^1^H NMR and ^13^C NMR data compared with previously published data [27,28]. Their purities were >95% as determined by HPLC-diode array detection (Figure S2a) and ^1^H NMR spectra (Figures S3 and S4).

### 4.3. Cell Culture

HepG2 cells, a human hepatoma-derived cell line, was obtained from ATCC (Manassas, VA, USA) and cultured in Dulbecco′s modified Eagle′s medium (DMEM) supplemented with 10% fetal bovine serum (FBS), 100 U/mL penicillin, and 100 μg/mL streptomycin in a humidified atmosphere of 5% CO_2_ at 37 °C. HepG2 cells that had been stably transfected with the pGL4.37 [luc2P/ARE/Hygro], an ARE-driven reporter gene construct, were kindly donated by Dr. IJ Cho (Daegu Haany University, Kyeongsan, Korea) [44]. Hygromycin was added to the cell culture medium to maintain pGL4.37 plasmid stably transfected HepG2 cells.

### 4.4. Cell Viability Assay

HepG2 cells were seeded at a density of 5 × 10^4^ cells/well in 48-well plates. To evaluate the cytotoxicity of pachypodol and eriodictyol 3′,7-dimethyl ether, the cells were serum-starved overnight and exposed to each compound for 24 h. To determine their effects on *t*-BHP-induced cell death, cells were preincubated with each compound for 1 h or 12 h after overnight serum starvation and subsequently exposed to 500 μM *t*-BHP for 4 h. The cell viability was measured by an MTT colorimetric assay [32]. Briefly, the cells were treated with 0.3 mg/mL MTT for the last 1 h at 37 °C and the media were then removed. Formazan crystals produced in each well were dissolved by adding 300 μL of DMSO and absorbance was measured at 570 nm with an Infinite M200 PRO microplate reader (Tecan, Salzburg, Austria).

### 4.5. Luciferase Assay

The pGL4.37 plasmid stably transfected HepG2 cells were seeded at a density of 2 × 10^5^ cells/well in 12-well plates and used for experiments when they were 70%–80% confluent. The cells were exposed to pachypodol and eriodictyol 3′,7-dimethyl ether for 12 h after overnight serum starvation and lysed with 1×passive lysis buffer (Promega, Madison, WI) after washing twice with ice-cold PBS. Luciferase activities in the cell lysates were measured using the luciferase assay system (Promega, Madison, WI, USA) in a luminometer (CentroPRO LB962; Berthold Technologies, Bad Wildbad, Germany).

### 4.6. Preparation of Nuclear Extracts and Western Blot Analysis

HepG2 cells were plated at a density of 5 × 10^5^ cells/well in 6-well plates, grown to reach 70–80% confluency, and then serum starved overnight before drug treatment. Nuclear extracts were prepared according to the method previously described [45]. Briefly, cells were washed and scraped from the dishes with PBS, and centrifuged at 2000 *g* for 5 min. Next, the cell pellets were allowed to swell by adding the hypotonic lysis buffer and then incubated on ice for 10 min. After centrifugation at 7200 *g* for 5 min at 4 °C, the pellets were resuspended in the extraction buffer and then incubated on ice for 1 h. The supernatants containing nuclear fractions were obtained after centrifugation at 15,000 *g* for 10 min. Western blot analyses were performed according to the previously published method [32]. Briefly, proteins in whole cell lysates or nuclear fractions were resolved by SDS-polyacrylamide gel electrophoresis and transferred onto nitrocellulose membrane (GE Healthcare, Chalfont St. Giles, Buckinghamshire, UK). After blocked with 5% (*w*/*v*) skim milk in PBS containing 0.25% (*v*/*v*) tween-20 for 1 h, the membrane was incubated with a primary antibody overnight at 4 °C and reacted with an HRP-conjugated secondary antibody (Life Technologies, Grand Island, NY, USA). ECL chemiluminescence detection kit (GE Healthcare, Chalfont St. Giles, Buckinghamshire, UK) was used to develop the protein bands. Equal loading of proteins were verified by immunoblotting for β-actin or lamin A/C.

### 4.7. Measurement of Intracellular ROS Generation

CM-H_2_DCFDA, a cell-permeable non-fluorescent probe was used as an indicator for the intracellular ROS. It is cleaved by intracellular esterases and CM-DCF, a fluorescent product is formed upon oxidation. HepG2 cells which had been exposed to *t*-BHP with or without pachypodol were subsequently incubated with 10 μM CM-H_2_DCFDA for the last 25 min at 37 °C. The cells were washed with PBS, fixed with 4% paraformaldehyde for 30 min, and then examined under a Nikon Eclipse Ti inverted fluorescence microscopy (Nikon, Tokyo, Japan). To quantify the fluorescence intensity, the cells were detached by trypsinization, washed with PBS, and resuspended in PBS supplemented with 1% FBS. The fluorescence intensity was measured using a FLUOstar Omega microplate reader (BMG Labtech, Offenburg, Germany) with excitation and emission wavelengths of 485 nm and 520 nm, respectively and was normalized to each protein concentration.

### 4.8. Measurement of Intracellular Glutathione (GSH) Content

HepG2 cell pellets were resuspended in ice-cold 5% (*w*/*v*) metaphosphoric acid solution and sonicated for 30 s. After ice incubation for 5 min, the cell suspension was centrifuged at 16,200 *g* for 5 min at 4 °C. The intracellular GSH content was determined in the supernatant by the colorimetric assay using a commercial kit (Bioxytech GSH-420; OxisResearch, Burlingame, CA, USA). Absorbance was measured at 400 nm with an Infinite M200 PRO microplate reader (Tecan, Salzburg, Austria).

### 4.9. Real-Time PCR Assay

Total RNA was extracted from HepG2 cells using Trizol (Invitrogen, Carlsbad, CA, USA) according to the manufacturer’s instructions. The isolated RNA (2 μg) was reverse transcribed to obtain cDNA by using oligo-d(T)_16_ primers, dNTP mix, and AMV Reverse Transcriptase (Promega, Madison, WI, USA) in SureCycler 8800 (Agilent Technologies, Santa Clara, CA). Real-time PCR analysis was conducted with LightCycler® 480 II instrument using LightCycler® 480 SYBR Green I Master solution (Roche Diagnostics, Indianapolis, IN, USA). A melting curve analysis was also performed to verify the accuracy of each amplicon. The glyceraldehyde-3-phosphate dehydrogenase (GAPDH) was used to normalize each mRNA expression. The following primers were used: human GCLC (sense: 5′-TGGCAATGCAGTGGTGGAT-3′, antisense: 5′-AACACACCTTCCTTCCCATTGA-3′); human GCLM (sense: 5′-GATCCAAAAGAACTGCTTTCTGAAG-3′, antisense: 5′- CCTCTACTTTTCACAATGACCGAAT-3′); human NQO1 (sense: 5′-AGGCTGGTTTGAGCGAGT-3′, antisense: 5′-ATTGAATTCGGGCGTCTGCTG-3′); human GAPDH (sense: 5′-GAAGGTGAAGGTCGGAGTC-3′, antisense: 5′-GAAGATGGTGATGGGATTTC-3′).

### 4.10. Statistical Analysis

All data are reported as mean ± S.D. Statistically significant differences were assessed by the Student’s *t*-test or one-way ANOVA with Bonferroni’s multiple comparison test. A probability value of less than 0.05 was considered significant.

## Supplementary Materials

Supplementary materials can be found at https://www.mdpi.com/1422-0067/20/17/4082/s1.

## Abbreviations

AMPK	AMP-activated protein kinase
ARE	Antioxidant response element
CM-H_2_DCFDA	5-(and-6)-Chloromethyl-2′,7′-dichlorodihydrofluorescein diacetate acetyl ester
CPC	Centrifugal partition chromatography
DMSO	Dimethyl sulfoxide
ERK	Extracellular signal-regulated kinase
GAPDH	Glyceraldehyde-3-phosphate dehydrogenase
GCL	Glutamate-cysteine ligase
GCLC	Catalytic subunit of glutamate-cysteine ligase
GCLM	Modifier subunit of glutamate-cysteine ligase
GSH	Glutathione
Keap1	Kelch-like ECH-associated protein 1
LPS	Lipopolysaccharide
MEK	Mitogen-activated protein kinase kinase
MTT	3-(4,5-Dimethylthiazol-2-yl)-2,5-diphenyltetrazolium bromide
Nrf2	NF-E2-related factor-2
NQO1	NAD(P)H:quinone oxidoreductase 1
PI3K	Phosphoinositide 3-kinase
ROS	Reactive oxygen species
*t*-BHP	*tert*-Butylhydroperoxide
