# Supplementary material for: Pachypodol, a Methoxyflavonoid Isolated from Pogostemon cablin Bentham Exerts Antioxidant and Cytoprotective Effects in HepG2 Cells: Possible Role of ERK-Dependent Nrf2 Activation
Source: Int J Mol Sci. 2019 Aug 21;20(17):4082. doi: 10.3390/ijms20174082 (PMC6747508; doi:10.3390/ijms20174082)
Supplement: Supplementary file 1 [file ijms-20-04082-s001.pdf]

Supplementary Materials

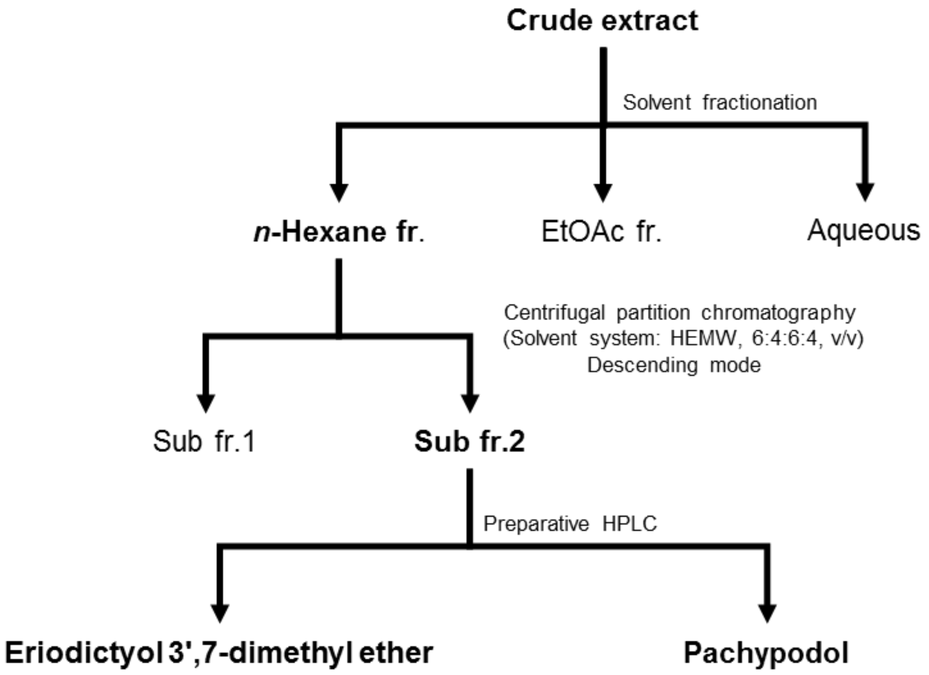

Figure S1. Activity-guided isolation scheme for pachypodol and eriodictyol 3',7-dimethyl ether from *P. cablin*.

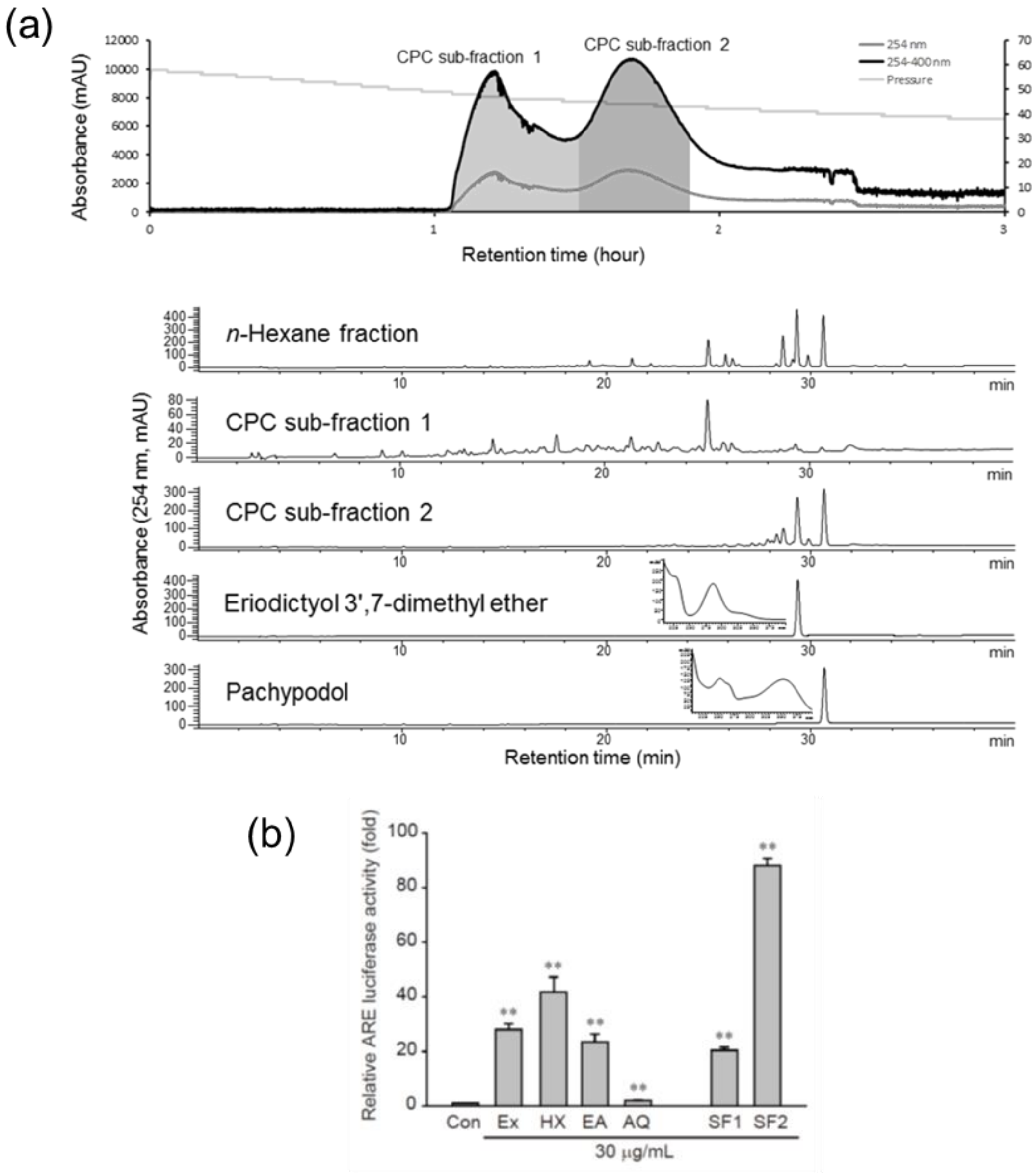

Figure S2. HPLC chromatograms and ARE-luciferase activities of crude extract and sub-fractions from *P. cablin*. (**a**) CPC chromatogram of the *n*-hexane fraction from *P. cablin* and HPLC chromatograms of CPC sub-fractions and isolated compounds. CPC operation conditions: two-phase solvent system, *n*-hexane–ethyl acetate–methanol–water with a volume ratio 6:4:6:4; descending mode, mobile phase-lower aqueous phase; flow-rate, 10 mL/min; rotation speed, 1200 rpm; monitored at 254 nm and scan mode (254–400 nm). HPLC conditions: Column, INNO Column C18 (4.6 x 250 mm, 5 μm, YoungJin Biochrom, Korea); mobile phase, acetonitrile (1% formic acid, solvent A)-water (1% formic acid, solvent B) in a gradient mode: 0-50 min, 10-100 % A; 60 min, 100 % A. The flow rate was 1 mL/min, whereas the injection volume was 10 μL. The diode array detector (DAD) measured UV spectrum over a range of 210 to 600 nm and the chromatogram of the effluents was recorded at 254 nm. (**b**) ARE-luciferase activities in the lysates of pGL4.37 plasmid stably transfected HepG2 cells treated with crude extract, each solvent fraction, or CPC sub-fractions for 12 h. Data represent the mean ± S.D. (n = 3). ***P*<0.01 (compared with the DMSO-treated vehicle control). AQ, aqueous; ARE, antioxidant response element; CPC, centrifugal partition chromatography; EA, ethyl acetate; Ex, crude extract; HX, *n*-hexane; SF1/2, CPC sub-fraction 1/2.

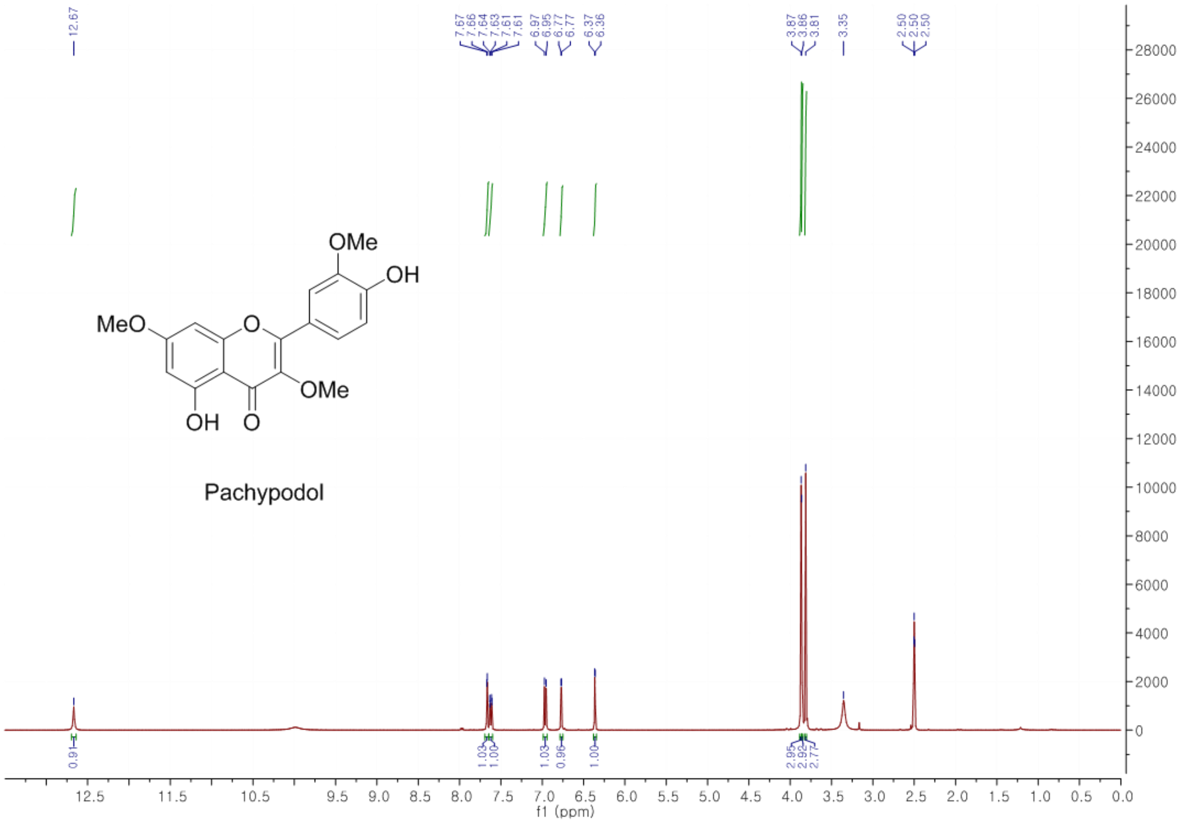

Figure S3. ^{1}H NMR spectrum of pachypodol. ^{1}H NMR (400 MHz, DMSO-*d*6) δ 12.67 (s, 1H, 5-OH), 7.67 (d, *J* = 1.9 Hz, 1H, H-2'), 7.62 (dd, *J* = 8.4, 1.9 Hz, 1H, H-6'), 6.96 (d, *J* = 8.4 Hz, 1H, H-5'), 6.77 (d, *J* = 2.1 Hz, 1H, H-6), 6.36 (d, *J* = 2.1 Hz, 1H, H-8), 3.87 (s, 3H, OMe), 3.86 (s, 3H, OMe), 3.81 (s, 3H, OMe).

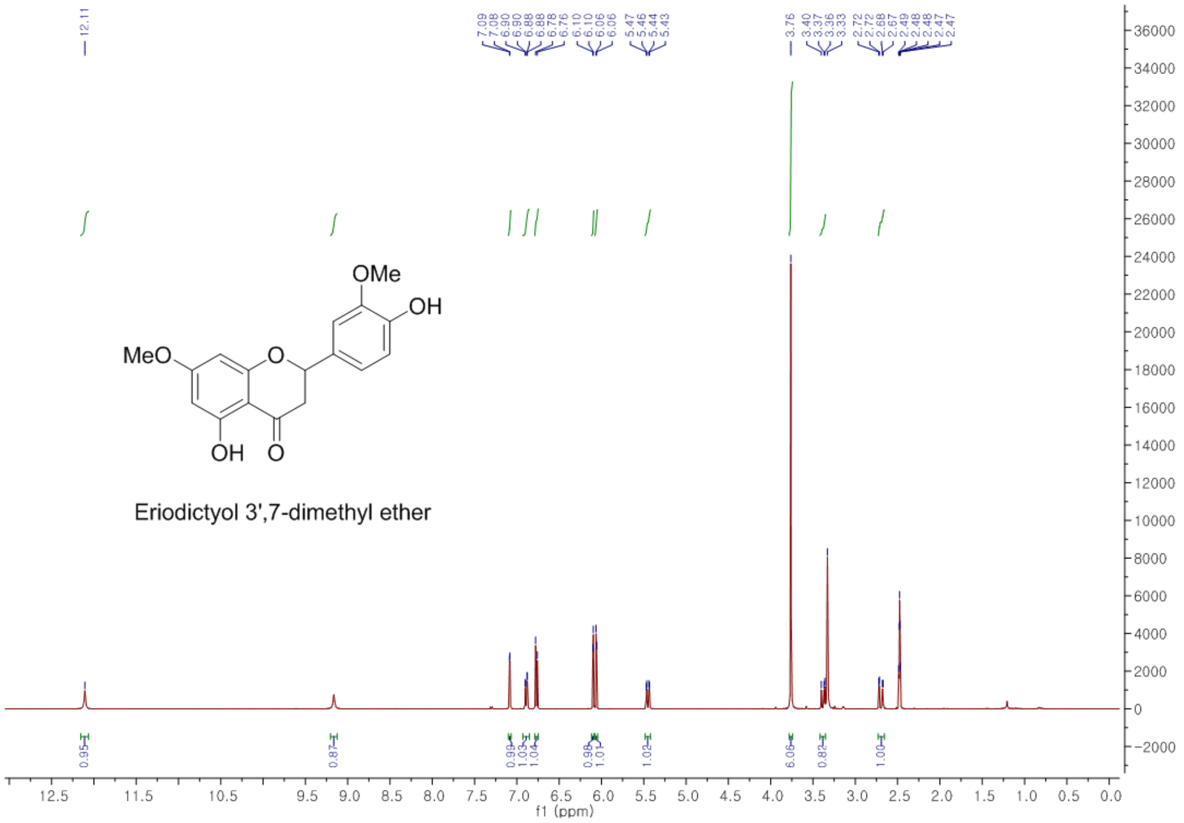

Figure S4. ^{1}H NMR spectrum of eriodictyol 3',7-dimethyl ether. ^{1}H NMR (400 MHz, DMSO-*d6*) δ 12.13 (s, 1H, 5-OH), 9.19 (s, 1H, 4'-OH), 7.11 (d, *J* = 1.9 Hz, 1H, H-2'), 6.91 (dd, *J* = 8.1, 1.9 Hz, 1H, H-6'), 6.79 (d, *J* = 8.1 Hz, 1H, H-5'), 6.12 (d, *J* = 2.3 Hz, 1H, H-8), 6.08 (d, *J* = 2.3 Hz, 1H, H-6), 5.47 (dd, *J* = 13.0, 2.8 Hz, 1H, H-2), 3.79 (s, 6H, OMe), 3.44 – 3.37 (m, 1H, $H\text{-}3_{trans}$), 2.72 (dd, *J* = 17.2, 3.0 Hz, 1H, $H\text{-}3_{cis}$).

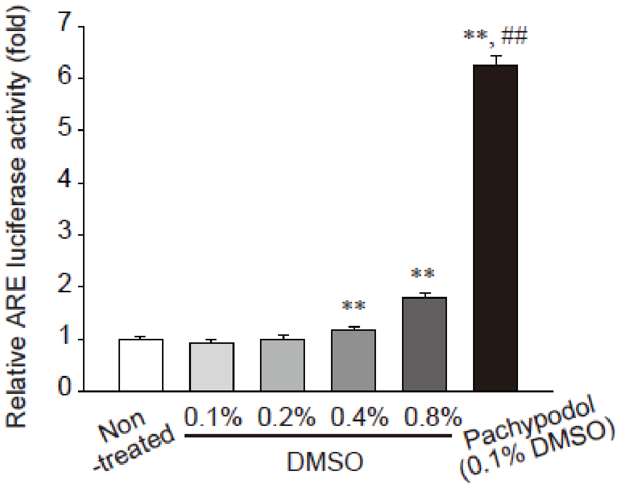

Figure S5. Effect of increasing concentrations of dimethyl sulfoxide (DMSO) on the Nrf2-ARE pathway in HepG2 cells. The ARE-luciferase activity was measured in the lysates of HepG2 cells stably transfected with pGL4.37 plasmid. Cells were non-treated or treated with either DMSO or 30 μM pachypodol for 12 h. Data represent the mean ± S.D. (n = 4). **P*<0.05, ***P*<0.01 (compared with the value in non-treated cells); ##*P*<0.01 (compared with the value in 0.1% DMSO-treated cells); ARE, antioxidant response element.
